# Selective Parathyroid Autotransplantation During Total Thyroidectomy for Papillary Thyroid Carcinoma: A Cohort Study

**DOI:** 10.3389/fsurg.2021.683041

**Published:** 2021-06-28

**Authors:** Yuxuan Qiu, Zhichao Xing, Yuanfan Qian, Yuan Fei, Yan Luo, Anping Su

**Affiliations:** ^1^Department of Ultrasound, West China Hospital, Sichuan University, Chengdu, China; ^2^Center of Thyroid & Parathyroid Surgery, West China Hospital, Sichuan University, Chengdu, China

**Keywords:** papillary thyroid carcinoma, parathyroid autotransplantation, hypoparathyroidism, parathyroid hormone, total thyroidectomy

## Abstract

**Purpose:** The relationship between the selective parathyroid gland (PG) autotransplantation and hypoparathyroidism is still not completely clear. The aim was to ascertain whether the number of autotransplanted PGs affected the incidence of hypoparathyroidism and recovery of parathyroid function in the long-term for patients with papillary thyroid carcinoma (PTC).

**Methods:** A retrospective cohort study included all patients with PTC who had underwent primary total thyroidectomy with central neck dissection between January 2013 and December 2017. The patients were divided into three groups (0, 1, and 2 PGs autotransplanted, respectively).

**Results:** Of the 2,477 patients, 634 (25.6%) received no PG autotransplantation, 1,078 (43.5%) and 765 (30.9%) were autotransplanted 1 and 2 PGs, respectively, and the incidence of permanent hypoparathyroidism (>1 year) was 1.7%, 0.7%, and 0.4% (*P* = 0.0228). Both 1 or 2 PGs autotransplanted increased the incidence of transient biochemical hypoparathyroidism (odds ratio [OR], 1.567; 95% confidence interval [CI], 1.258–1.953; *P* < 0.0001; OR, 2.983; 95% CI, 2.336–3.810; *P* < 0.0001, respectively) but reduced the incidence of permanent hypoparathyroidism (OR, 0.373; 95% CI, 0.145–0.958; *P* = 0.0404; OR, 0.144; 95% CI, 0.037–0.560; *P* = 0.0052, respectively). Both 1 or 2 PGs autotransplanted did not independently influence the occurrence of hypocalcemia symptoms.

**Conclusion:** Selective parathyroid autotransplantation is less likely to lead to post-operative symptomatic hypocalcemia, although it could lead to a transient decrease in parathyroid hormone. However, in the long run, it is still an effective strategy to preserve parathyroid function.

## Introduction

The treatment of the rapidly increasing incidence of papillary thyroid carcinoma (PTC) has led to the widespread use of total thyroidectomy with central neck dissection (CND) (1). However, this surgical treatment increases the risk of post-operative hypoparathyroidism, especially when bilateral central neck dissection is performed (2). When a low intact parathyroid hormone (PTH) level is accompanied by hypocalcemia, hypoparathyroidism will occur (3). Although no agreement for diagnostic criteria of hypoparathyroidism was reached, the former reported incidence of permanent hypoparathyroidism ranges from 0.0 to 20.2%, with a median of 2.0% (4, 5).

Ideally, preservation of every parathyroid gland (PG) *in situ* with its blood supply is the best method, but this is practically challenging, even for high-volume surgeons (6). Although meticulous dissection is feasible, PGs can still become devascularized, and are occasionally found in the surgical specimens (7). Therefore, the autotransplantation of devascularized or unintentionally removed PGs is most commonly adopted, known as selective PG autotransplantation (8). However, some authors even advocated routine PG autotransplantation due to their belief that the function of the autotransplanted PGs is more predictable than the PGs which is left *in situ* with a possibly insufficient blood supply, which would reduce the incidence of hypoparathyroidism (9). Although the demonstration of graft function has been obtained in several studies using a peripheral site for PG autotransplantation (10), there is still a lack of a series of consistent direct trials. The association between the number of autotransplanted PGs and recovery of parathyroid function is still controversial.

Therefore, the aim of this study was to determine whether selective PG autotransplantation contributes to the recovery of parathyroid function in long-term follow-up, based on the inpatients who received total thyroidectomy for PTC in our center in the recent 5 years.

## Materials and Methods

The study was conducted in accordance with the guideline of Strengthening the Reporting of Observational Studies in Epidemiology (STROBE) Statement for cohort studies (11).

### Patients

We conducted a retrospective cohort study, including all patients with papillary thyroid carcinoma (PTC) who underwent primary total thyroidectomy in our center from January 2013 to December 2017. Exclusion criteria included age <18 years old, severe chronic renal insufficiency, perioperative hyperparathyroidism or hypoparathyroidism, reoperation, endoscopic thyroidectomy, lobectomy, completion thyroidectomy, and lack of follow-up for at least 2-year. Tumors were divided into different stages according to the American Joint Committee for Cancer (AJCC) staging system (8th edition) (12). Patients were divided into three groups: group0 (without autotransplantation), group1 (autotransplantation of 1 PG), and group2 (autotransplantation of 2 PGs). The research was approved by the medical ethics committee of West China Hospital, Sichuan University. All patients who participated in this study obtained the informed consent, that is their clinical data could be used for medical analysis.

### Indications of Total Thyroidectomy With Lymph Node Dissection

The indications for total thyroidectomy, instead of lobectomy were as follows: (1) high risk radiation exposure or family history; (2) bilateral or multifocal PTC; (3) unilateral PTC with contralateral thyroid nodule(s); (4) isthmus PTC; (5) PTC with a maximum diameter of over 4 cm (stage T3a); (6) PTC with extrathyroidal extension (stages T3b and T4); (7) high risk pathologic variants including tall cell variant, diffuse sclerosis variant, and solid variant; (8) PTC with bilateral central neck lymph node or lateral neck lymph node metastases; (9) PTC with distant metastases; and (10) TERT promoter mutation (confirmed by pre-operative fine-needle aspiration biopsy). Unilateral central neck dissection was performed routinely, unless bilateral central neck dissection is indicated because of (1) bilateral PTC; (2) isthmus PTC; (3) PTC that staged T3 or T4; (4) prelaryngeal and/or pretracheal lymph node metastases; (5) bilateral central lymph node or lateral lymph node metastases; and (6) TERT promoter mutation (conformed by pre-operative fine-needle aspiration biopsy). Therapeutic lateral neck dissection was performed in the patients whose lateral lymph node metastasis was confirmed by pre-operative fine-needle aspiration biopsy (7).

### Surgical Procedures

All surgeries were performed by four surgeons with similar operation level and style in our center. Carbon nanoparticles were recommended for each patient for better identification of PGs, but the use of them was finally dependent on the patient's will. Generally, thyroid or lymph tissues can be stained black by carbon nanoparticles while parathyroid glands cannot because of their different lymphatic drainage. After first injection of carbon nanoparticles, every effort was made to identify each PG and carefully preserve its blood supply, so as to ensure this PG was preserved *in situ* with sufficient blood supply. When a PG was devascularized or resected unintentionally, some tissue of this PG would be examined by intraoperative frozen section biopsy. If confirmed, this PG would be chopped into 1mm^3^ fragments and autografted into the contralateral sternocleidomastoid muscle. The same group of pathologists analyzed all surgical specimens and the presence of PGs in the surgical specimens was recorded (7, 13).

### Perioperative Management

Perioperative management of all patients was standardized. Pre-operative examinations included serum calcium, PTH, thyroid function, neck ultrasound, and laryngoscopy. Symptomatic hypocalcemia is defined as serum calcium is lower than the normal limit (normal range, 2.1–2.7 mmol/L), accompanied by hypocalcemia manifestations. If a patient had symptomatic hypocalcemia, oral or intravenous calcium supplementation would be added and prolonged. Serum PTH and calcium levels were also measured on the first day after surgery.

### Follow-Up and Hypoparathyroidism

Routine laboratory tests, including serum PTH and calcium levels, were performed at 1 month, 3 months, 6 months, 12 months, and 2 years after surgery. ^131^I ablation is performed by department of nuclear medicine within 2–3 months after surgery, if necessary. Biochemical transient hypoparathyroidism was defined as any drop only in serum PTH below the normal limit (normal range, 1.6–6.9 pmol/L) post-operatively and recovery to the normal limit within 6 months, while clinical transient hypoparathyroidism is combined with both reduced serum PTH below the normal range and symptomatic hypocalcemia which needed added and prolonged oral or intravenous calcium supplementation. If serum PTH was lower than the normal limit with symptomatic hypocalcemia, but recovered to normal between 6 to 12 months after surgery, it would be defined as protracted hypoparathyroidism. Permanent hypoparathyroidism was defined as lack of recovery of serum PTH to the normal range within 12 months, with accompanied symptomatic hypocalcemia.

### Statistical Analysis

The statistical analyses were carried out using STATA 15.1. Continuous variables were expressed as mean ± standard deviation (SD). The χ^2^ test was used to evaluate the differences of incidences, and the analysis of variance (ANOVA) was used to evaluate the differences of continuous variables. Logistic regression was conducted to confirm how number of PG autotransplantation affects hypoparathyroidism, by unadjusted model and model adjusted by variables including gender, age, pre-operative PTH levels, surgical extent, if PG/s unintentionally resected, and if carbon nanoparticles were used. The results were expressed as odds ratio (OR) and 95% confidence interval (CI).

## Results

### Patient Characteristics

**Figure 1:**
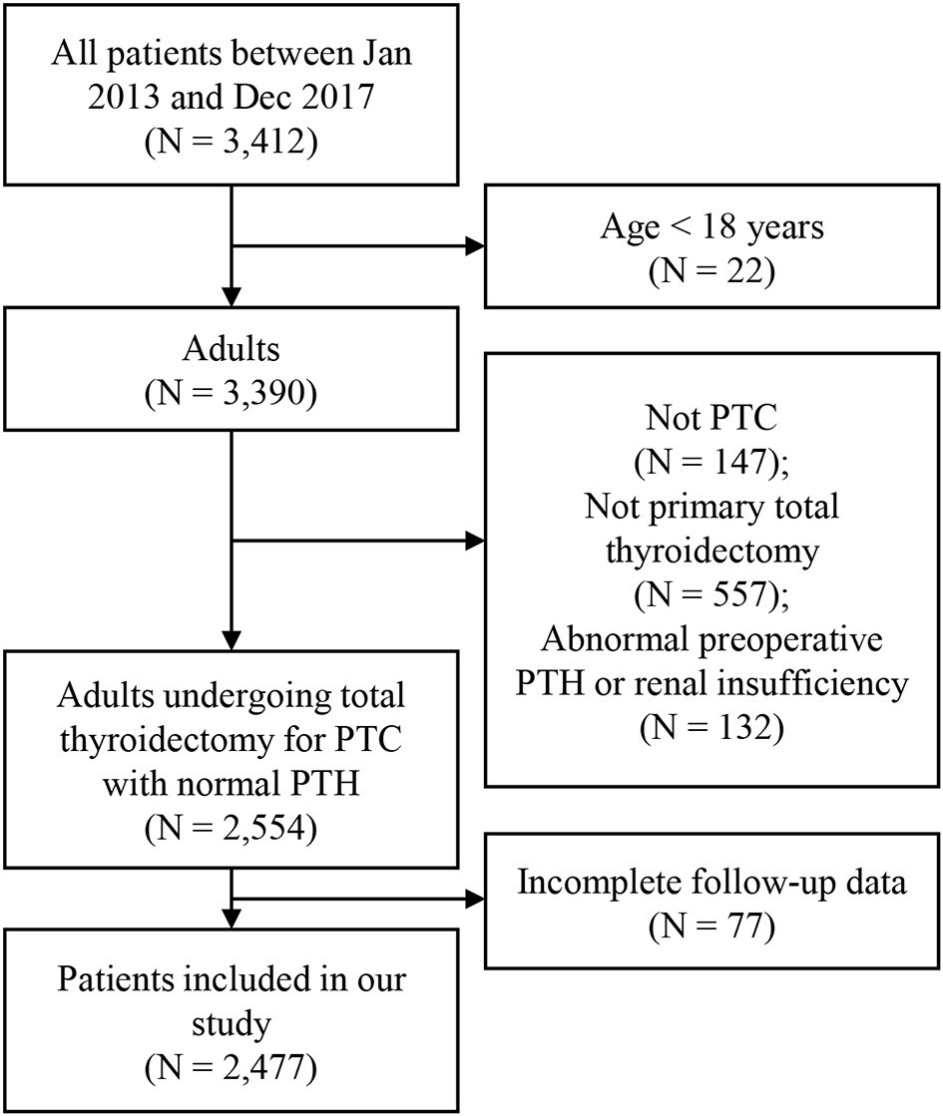
Flow chart of included patients.

A total of 2,477 patients met the inclusion criteria of our study. Figure 1 showed the inclusion flow path. There were 645 (26.0%) male and 1,832 (74.0%) female patients, ranging from 18 to 83 years old, with an average of 42.60 ± 11.80 years old. All patients received total thyroidectomy; 631 (25.5%) had unilateral CND, and 1,846 (50.8%) had bilateral CND, with or without lateral neck dissection. The number of PGs autotransplanted was as follows: 0 in 634 (25.6%) patients (group0); 1 in 1078 (43.5%) patients (group1); 2 in 765 (30.9%) patients (group2). Table 1 showed the characteristics of the three groups of patients. There was significant difference in age distribution (older in group0; *P* = 0.0233), however, there was no difference among three groups in patients aging ≥ 55 years (*P* = 0.8177). With increasing number of autotransplanted PGs, more severe N stage were found (*P* = 0.0011). All other characteristics were not different among the three groups.

**Table 1:** Basic characteristics of included patients.

**Variables**		**Group0 (*N* = 648) *N* (%)**	**Group1 (*N* = 1078) *N* (%)**	**Group2 (*N* = 765) *N* (%)**	***P***
Gender	Male	182 (28.7)	265 (24.6)	198 (25.9)	0.1704
	Female	452 (71.3)	813 (75.4)	567 (74.1)	
Age	Mean ± SD	43.71 ± 11.96	42.23 ± 11.87	42.20 ± 11.50	0.0233^*^
	<55	536 (84.5)	918 (85.2)	656 (85.8)	0.8177
	≥55	98 (15.5)	160 (14.8)	109 (14.2)	
BMI	Mean ± SD	23.20 ± 3.31	23.27 ± 3.91	23.23 ± 3.25	0.9329
	<24	398 (63.3)	658 (62.0)	448 (60.1)	0.4584
	24	231 (36.7)	403 (38.0)	298 (39.9)	
Hospitalization (day)	Mean ± SD	8.41 ± 3.55	8.62 ± 3.51	8.49 ± 2.59	0.4365
Hypertension	No	562 (88.6)	975 (90.4)	697 (91.1)	0.2827
	Yes	72 (11.4)	103 (9.6)	68 (8.9)	
Diabetes	No	613 (96.7)	1052 (97.6)	739 (96.6)	0.3828
	Yes	21 (3.3)	26 (2.4)	26 (3.4)	
Hypothyroidism	No	619 (97.6)	1055 (97.9)	745 (97.4)	0.7967
	Yes	15 (2.4)	23 (2.1)	20 (2.6)	
Grave's disease	No	614 (96.8)	1049 (97.3)	746 (97.5)	0.7385
	Yes	20 (3.2)	29 (2.7)	19 (2.5)	
HD	No	495 (78.1)	813 (75.4)	599 (78.3)	0.2634
	Yes	139 (21.9)	265 (24.6)	166 (21.7)	
NG	No	261 (41.2)	472 (43.8)	347 (45.4)	0.2859
	Yes	373 (58.8)	606 (56.2)	418 (54.6)	
Upper lobe	No	455 (71.8)	764 (70.9)	562 (73.5)	0.4734
	Yes	179 (28.2)	314 (29.1)	203 (26.5)	
Isthmus	No	588 (92.7)	994 (92.2)	706 (92.3)	0.9168
	Yes	46 (7.3)	84 (7.8)	59 (7.7)	
Bilaterality	No	536 (84.5)	923 (85.6)	631 (82.5)	0.1864
	Yes	98 (15.5)	155 (14.4)	134 (17.5)	
Multifocality	No	477 (75.2)	809 (75.0)	553 (72.3)	0.3294
	Yes	157 (24.8)	269 (25.0)	212 (27.7)	
ETE	No	549 (86.6)	892 (82.7)	652 (85.2)	0.0836
	Yes	85 (13.4)	186 (17.3)	113 (14.8)	
Largest tumor size (mm)	Mean ± SD	13.37 ± 9.15	13.65 ± 9.4	14.15 ± 9.59	0.3026
T stage	Tx	5 (0.8)	2 (0.2)	3 (0.4)	0.0533
	T1a	276 (43.5)	457 (42.4)	315 (41.2)	
	T1b	214 (33.8)	337 (31.3)	238 (31.1)	
	T2	47 (7.4)	81 (7.5)	86 (11.2)	
	T3a	5 (0.8)	14 (1.3)	10 (1.3)	
	T3b	54 (8.5)	116 (10.8)	67 (8.8)	
	T4a	30 (4.7)	69 (6.4)	46 (6.0)	
	T4b	3 (0.5)	2 (0.2)	0 (0)	
N stage	Nx	6 (0.9)	7 (0.6)	4 (0.5)	0.0011^*^
	N0	335 (52.8)	534 (49.5)	336 (43.9)	
	N1a	197 (31.1)	336 (31.2)	240 (31.4)	
	N1b	96 (15.1)	201 (18.6)	185 (24.2)	

*HD, Hashimoto's disease; NG, nodular goiter; ETE, extrathyroidal extension; SD, standard deviation*;

* *P < 0.05*.

### Parathyroid Related Characteristics and Follow-Up

**Figure 2:**
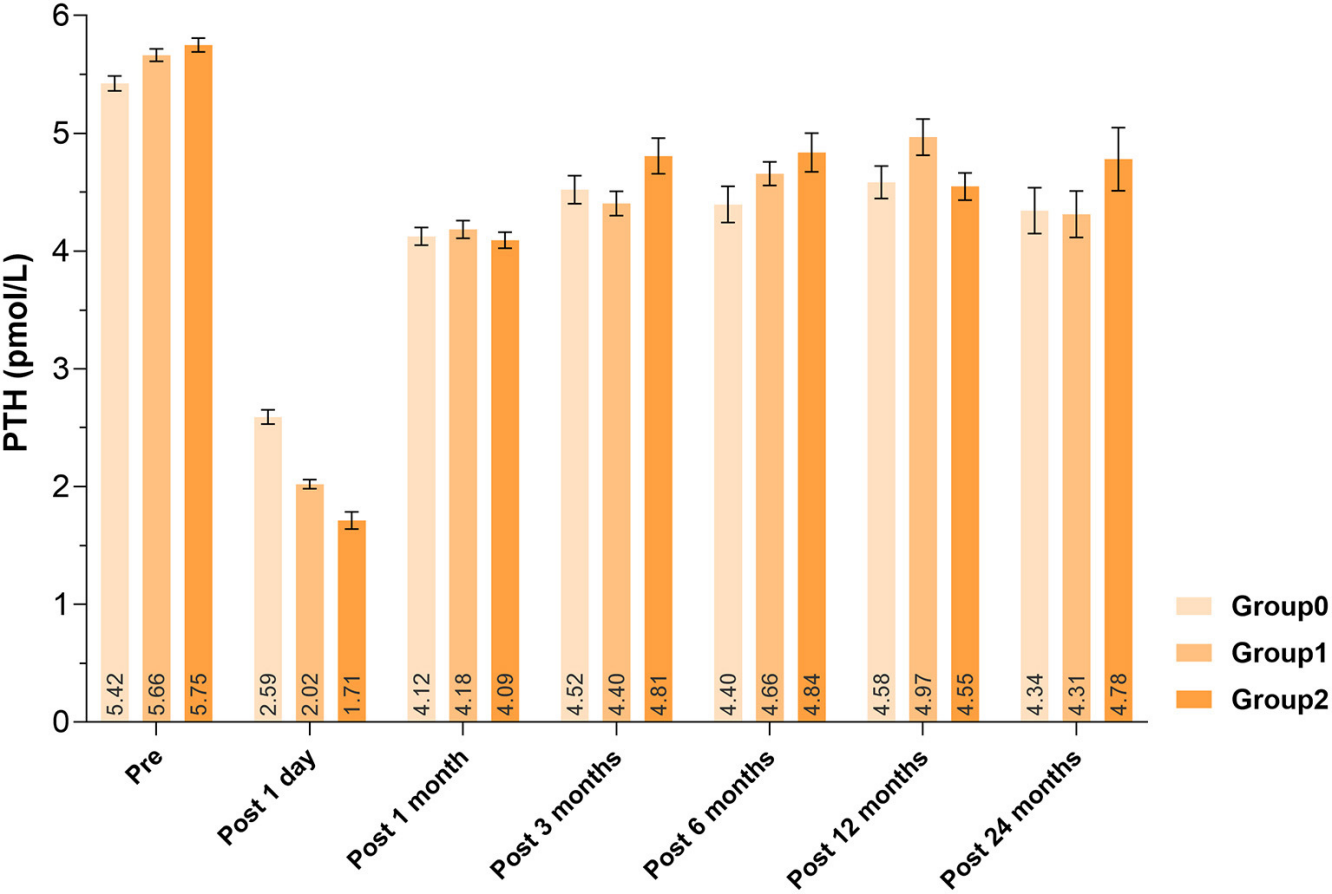
Changes of parathyroid hormones among groups. Pre, pre-operative; Post, post-operative.

Patients with increasing number of autotransplanted PGs underwent more extensive lymph node dissection, showing more bilateral CND (*P* < 0.0001). Less use of carbon nanoparticles (*P* < 0.0001) and more unintentionally resected PGs (*P* = 0.0019) were also associated with more PGs autotransplanted (Table 2). About the laboratory test on 1 day after surgery, lower PTH levels (*P* < 0.0001) and calcium levels (P = 0.0061) were detected, with the increase number of autotransplanted PGs. However, there was no significant difference of PTH or calcium levels until end of follow-up (*P* < 0.05). Comparisons of serum PTH levels were displayed in Figure 2.

**Table 2:** Parathyroid related characteristics of included patients.

**Variables**		**Group0 (*N* = 634) N (%)**	**Group1 (*N* = 1,078) *N* (%)**	**Group2 (*N* = 765) *N* (%)**	***P***
Carbon nanoparticles	No	119 (18.8)	278 (25.8)	414 (54.1)	<0.0001^*^
	Yes	515 (81.2)	800 (74.2)	351 (45.9)	
Surgical extent	Unilateral CND	282 (44.5)	286 (26.5)	63 (8.2)	<0.0001^*^
	≥Bilateral CND	352 (55.5)	792 (73.5)	702 (91.8)	
PG unintentionally resected	No	595 (93.8)	978 (90.7)	676 (88.4)	0.0019^*^
	Yes	39 (6.2)	100 (9.3)	89 (11.6)	
Vitamin D deficiency	No	240 (37.9)	333 (30.9)	248 (32.4)	0.0111^*^
	Yes	394 (62.1)	745 (69.1)	517 (67.6)	
PTH, pmol/L (Mean ± SD)	Pre	5.42 ± 1.61	5.66 ± 1.70	5.75 ± 1.63	0.0008^*^
	Post 1 day	2.59 ± 1.55	2.02 ± 1.28	1.71 ± 2.01	<0.0001^*^
	Post 1 month	4.12 ± 1.76	4.18 ± 2.30	4.09 ± 1.80	0.6454
	Post 3 months	4.52 ± 1.82	4.40 ± 2.17	4.81 ± 2.72	0.0515
	Post 6 months	4.40 ± 2.29	4.66 ± 2.11	4.84 ± 3.00	0.1183
	Post 12 months	4.58 ± 1.81	4.97 ± 2.74	4.55 ± 1.61	0.0676
	Post 24 months	4.34 ± 1.67	4.31 ± 2.34	4.78 ± 2.14	0.3242
Ca, mmol/L (Mean ± SD)	Pre	2.35 ± 0.42	2.33 ± 0.17	2.34 ± 0.13	0.6515
	Post 1 day	2.13 ± 0.15	2.11 ± 0.17	2.10 ± 0.16	0.0061
	Post 1 month	2.31 ± 0.16	2.3 ± 0.15	2.31 ± 0.13	0.7692
	Post 3 months	2.27 ± 0.16	2.26 ± 0.15	2.26 ± 0.15	0.3140
	Post 6 months	2.28 ± 0.15	2.26 ± 0.19	2.26 ± 0.14	0.0725
	Post 12 months	2.28 ± 0.15	2.28 ± 0.13	2.28 ± 0.17	0.8099
	Post 24 months	2.30 ± 0.16	2.27 ± 0.17	2.27 ± 0.11	0.2429
25-OH-VD, mmol/L (Mean ± SD)	Pre	46.83 ± 16.51	43.30 ± 15.76	45.04 ± 15.75	0.0352^*^
	Post 1 day	37.10 ± 14.71	32.95 ± 13.50	35.57 ± 16.40	0.0175^*^
	Post 1 month	50.33 ± 17.00	48.38 ± 16.77	50.55 ± 14.81	0.0280^*^
	Post 3 months	54.39 ± 16.32	51.10 ± 16.29	49.42 ± 16.01	0.0085^*^
	Post 6 months	57.22 ± 19.56	53.70 ± 18.80	52.11 ± 29.52	0.0989
	Post 12 months	60.29 ± 21.66	56.33 ± 21.05	58.26 ± 16.50	0.2820
	Post 24 months	70.04 ± 26.18	65.30 ± 18.48	58.56 ± 21.30	0.1646
Biochemical transient hypoparathyroidism	Yes	168 (26.5)	413 (38.3)	437 (57.1)	<0.0001
Clinical transient hypoparathyroidism	Yes	40 (6.3)	81 (7.5)	66 (8.6)	0.2628
Protracted hypoparathyroidism	Yes	14 (2.2)	16 (1.5)	8 (1.0)	0.2088
Permanent hypoparathyroidism	Yes	11 (1.7)	8 (0.7)	3 (0.4)	0.0228^*^

*CND, central neck dissection; Pre, pre-operative; Post, post-operative; SD, standard deviation*;

* *P < 0.05*.

### PG Autotransplantation and Risk of Hypoparathyroidism

A total of 1,205 (48.6%) patients developed transient hypoparathyroidism, in which 1,018 (41.1%) patients were biochemical hypoparathyroidism and 187 (7.5%) patients were clinical hypoparathyroidism. In addition, 38 (1.5%) patients were defined as protracted hypoparathyroidism, and 22 (0.9%) patients did not recover from reduced PTH levels and symptomatic hypocalcemia within 12 months. They were defined as permanent hypoparathyroidism (Table 2). In univariate analysis, 1 PG autotransplanted increased incidence of biochemical transient hypoparathyroidism (OR, 1.723; 95% CI, 1.390–2.136; *P* < 0.0001), and 2 PGs autotransplanted were associated with higher incidence of biochemical transient hypoparathyroidism (OR, 3.696; 95% CI, 2.945–4.638; *P* < 0.0001) and lower incidence of permanent hypoparathyroidism (OR, 0.223; 95% CI, 0.062–0.803; *P* = 0.0217). After adjusting for factors of clinical significance including gender, age, pre-operative vitamin D deficiency, surgical extent, PG/s unintentionally resected, and use of carbon nanoparticles, and factors with a *P*-value <0.15 in univariate analysis including extrathyroidal extension, T stage, and N stage, 1 PG autotransplanted increased incidence of transient biochemical hypoparathyroidism (OR, 1.567; 95% CI, 1.258–1.953; *P* < 0.0001) and decrease incidence of permanent hypoparathyroidism (OR, 0.373; 95% CI, 0.145–0.958; *P* = 0.0404), and 2 PGs autotransplanted were associated with higher incidence of transient biochemical hypoparathyroidism (OR, 2.983; 95% CI, 2.336–3.810; *P* < 0.0001) and lower incidence of permanent hypoparathyroidism (OR, 0.144; 95% CI, 0.037–0.560; *P* = 0.0052), showing in Table 3. In addition, all other independent risk factors for hypoparathyroidism were displayed in Table 4.

**Table 3:** Parathyroid autotransplantation and risk of hypoparathyroidism.

	**Number of autotransplanted PGs**	**Unadjusted OR (95% CI)**	***P***	**Adjusted OR (95% CI)**	***P***
Biochemical transient hypoparathyroidism	1 PG	1.723 (1.390–2.136)	<0.0001^*^	1.567 (1.258–1.953)	<0.0001^*^
	2 PGs	3.696 (2.945–4.638)	<0.0001^*^	2.983 (2.336–3.810)	<0.0001^*^
Clinical transient hypoparathyroidism	1 PG	1.206 (0.815–1.786)	0.3482	1.107 (0.742–1.652)	0.6190
	2 PGs	1.402 (0.933–2.108)	0.1042	1.236 (0.795–1.920)	0.3470
Protracted hypoparathyroidism	1 PG	0.667 (0.323–1.376)	0.2734	0.633 (0.302–1.329)	0.2267
	2 PGs	0.468 (0.195–1.123)	0.0890	0.394 (0.152–1.017)	0.0543
Permanent hypoparathyroidism	1 PG	0.423 (0.169–1.058)	0.0660	0.373 (0.145–0.958)	0.0404^*^
	2 PGs	0.223 (0.062–0.803)	0.0217^*^	0.144 (0.037–0.560)	0.0052^*^

*OR, odds ratio; CI, confidence interval*;

* *P < 0.05*.

**Table 4:** Clinical factors significantly associated with hypoparathyroidism in multivariate analysis.

	**Factor**	**Adjusted OR (95% CI)**	***P***
Biochemical transient	≥Bilateral CND	1.539 (1.246–1.900)	<0.0001^*^
hypoparathyroidism	PG unintentionally resected	1.777 (1.333–2.369)	<0.0001^*^
Clinical transient	Female	2.305 (1.505–3.531)	<0.0001^*^
hypoparathyroidism	Age ≥55	1.554 (1.064–2.271)	0.0227^*^
	≥Bilateral CND	1.562 (1.040–2.344)	0.0314^*^
Protracted hypoparathyroidism	–	–	–
Permanent hypoparathyroidism	Carbon nanoparticles	0.403 (0.167–0.972)	0.0432^*^
	PG unintentionally resected	4.061 (1.426–11.568)	0.0087^*^

*CND, central neck dissection; PG, parathyroid gland; OR, odds ratio; CI, confidence interval*;

* *P < 0.05*.

## Discussion

Since thyroidectomy became a common surgical method in the 1980's, post-operative complications have plagued most surgeons. Post-operative hypoparathyroidism and secondary hypocalcemia are the most common significant complications after thyroidectomy. Various factors may lead to hypoparathyroidism, especially accidental PG resection and PG autotransplantation (14–16). There is no doubt that PG autotransplantation is an effective method to restore PGs after inadvertent removal or devascularization (10, 17), and many researchers have demonstrated the long-term survival of the majority of parathyroid grafts (18, 19).

In our study, among 2,477 patients, 48.6% had transient hypoparathyroidism (41.1% biochemical and 7.5% clinical), 1.5% had protracted hypoparathyroidism, and 0.9% had permanent hypoparathyroidism. As the increase of number of autotransplanted PGs, the incidence of transient hypoparathyroidism increased but permanent hypoparathyroidism decreased, while protracted hypoparathyroidism had no difference. In addition, our study demonstrated that autotransplanted 1 PG and 2 PGs both independently increased the risk of transient hypoparathyroidism but reduced the risk of permanent hypoparathyroidism.

PG autotransplantation is an independent risk factor for transient hypoparathyroidism, which is beyond doubt and has been confirmed by many previous studies (16, 20, 21). When more PGs are autotransplanted, the fewer PGs remaining *in situ* might not sustain the normal PTH level because of the delayed functioning of the grafts. Su et al. reported that with the increase of number of autotransplanted glands, the incidence of transient hypoparathyroidism was 26.1% without PG autotransplanted, 36.2% for 1 PG autotransplanted, 52.6% for 2 PGs autotransplanted, and 84.6% for 3 PGs autotransplanted (*P* < 0.05) (7).

Parathyroid autotransplantation is often recommended to prevent permanent hypoparathyroidism. Some studies have found that autotransplantation of at least 1 PG could reduce or eliminate permanent hypoparathyroidism (9, 22), but this was not confirmed by other studies (10, 23). Palazzo et al. reported that the incidence of permanent hypoparathyroidism was 0.98, 0.77, 0.97, and 0% for none, 1, 2, and 3 PGs autotransplanted, respectively (24). Kihara et al. found that there was a positive correlation between incidence of permanent hypoparathyroidism and the number of autotransplanted PGs (25). However, owing to huge variations of definitions of permanent hypoparathyroidism, especially for cut-off time between transient and permanent hypoparathyroidism defining as 6 months, the real incidence of permanent hypoparathyroidism might be overestimated. We used 1 year as the definition of permanent hypoparathyroidism, not 6 months to observe the long-term outcomes of post-operative hypoparathyroidism. We found that compared with keeping all PGs *in situ*, PG autotransplantation was an independent protective factor for permanent hypoparathyroidism, which is true for both 1 PG or 2 PGs autotransplantation. Though our results recommended PG autotransplantation for preventing permanent hypoparathyroidism, whether routinely autotransplant PG should be recommended was still controversial. It reported that if all PGs autotransplanted, 21.4% of the patients would carry permanent hypoparathyroidism (26), and it indicated it is not suitable for completely relying on PG autotransplantation for preventing permanent hypoparathyroidism. Carefully preserving PGs *in situ* and selectively autotransplanting resected or devascularized PGs should still be adopted, but forcing to preserve all PGs *in situ* without considering injury or blood supply should be avoided.

Serum PTH concentration can be used as an index for the recovery of parathyroid function. Our study found that PTH levels decreased significantly with more PGs autotransplanted 1 day after surgery, and remained almost within the normal range from 1 month after surgery, which corresponded to the rule that autotransplanted PGs could not recover in the short term (8). El-Sharaky et al. found that post-operative parathyroid function of autotransplanted PGs would gradually recover from the 2nd week to a normal level at the 4th week, following blood tests and electron microscopy analysis (27). Qiu et al. found, out of the 964 patients, 23 (2.39%) developed permanent hypoparathyroidism and 105 (10.89%) recovered: 86 (8.92%) before 6 months, 11 (1.14%) within 6 and 12 months and 8 (0.83%) after 1 year follow-up; number of autotransplanted parathyroid glands (hazard ratio, 1.399; 95% CI, 1.060–1.846; *P* = 0.018) was significantly associated with the time to parathyroid function recovery (28). Promberger et al. found serum PTH concentrations in patients with 2 PGs autotransplanted recovered to 108% of the baseline levels at 6-month follow-up (29). In another study, the 5-year average post-operative recovery rates of without and with autotransplantation were 102 and 107%, respectively (25). However, in our study, after 2 years of follow-up, the PTH levels did not recovered to pre-operative level and we speculate that this might be due to mechanical or thermal injury to PGs both preserved *in situ* and autotransplanted during operation, actual dysfunction of PGs preserved *in situ* (it only looked intact), and dysfunction of the parathyroid grafts for inadequate blood supply or slow fibrosis.

There are several major limitations in this study. The data came from retrospective chart review. Then, the recovery of post-operative serum PTH levels depended on both the *in situ* preserved PGs and autotransplanted PGs, and may be the *in situ* preserved PGs were the main barrier against permanent hypoparathyroidism. Therefore, our further study can assess the function of autotransplanted glands by put them into forearm subcutaneous or muscle tissues, which could directly check the graft function by comparing serum PTH levels of the two arms (30). Another limitation is that the surgeon's judgment of devascularization is subjective, which might lead to the selection bias.

## Conclusion

In conclusion, PG autotransplantation is an effective strategy for mid- or long-term parathyroid function recovery for patients who received total thyroidectomy for PTC. Selective PG autotransplantation is an independent risk factor for transient hypoparathyroidism but a preventive factor for permanent hypoparathyroidism (>1 year). More number of autotransplanted PGs will help the parathyroid function recovery, if autotransplantation is really needed.

## Data Availability Statement

The raw data supporting the conclusions of this article will be made available by the authors, without undue reservation.

## Ethics Statement

The studies involving human participants were reviewed and approved by the ethic committee of West China Hospital, Sichuan University. The patients/participants provided their written informed consent to participate in this study.

## Author Contributions

YQiu and AS conceived and designed the study. YQiu, ZX, and YQian executed the study. YQiu, ZX, and YF analyzed and involved in interpretation of data. YQiu and ZX drafted the article. YQiu, YL, and AS made final approval of the version to be published. All authors contributed to the article and approved the submitted version.

## Conflict of Interest

The authors declare that the research was conducted in the absence of any commercial or financial relationships that could be construed as a potential conflict of interest.
